# Peptone Injections in Hæmoglobinuria

**Published:** 1910-05-28

**Authors:** 


### PEPTONE INJECTIONS IN HÆMOGLOBINURIA.

Both in purpuric conditions, such as Henoch's purpura, and in conditions of hæmoglobinuria benefit has been obtained from the administration, especially hypodermically, of horse serum. The main disadvantage in using the latter therapeutically, however, is connected with what is known as "anaphylaxis"; that is to say, that the injections of horse serum cannot be repeated after 10 days from the first injection without untoward results ensuing, owing to some change having taken place in the patient's body, rendering him extremely susceptible to serum poisoning. It has occurred to Dr. Nolf, of Liege, however, that by using some other proteid than horse serum the benefits of the latter might be obtained without any anaphylactic ill-effects; he has employed pro-peptone in the treatment of a case of paroxysmal hæmoglobinuria in which the attacks were brought on by any exposure to cold. He found the results to be good. The following is an account of the case

A miner, aged 34, who had never suffered from venereal trouble and who had not been ill previously, so far as he knew, came under observation in hospital for acute hæmoglobinuria. The first bout of it had occurred 14 months previously. It was followed by others at intervals of four, six, or eight weeks, and each attack was produced when the patient had remained exposed to the cold for a length of time. If the chill was considerable, the man experienced malaise, a degree of lassitude that amounted to a sense of bruising of his muscles, and he passed red urine for several hours. If the degree of chill was slight, such, for example, as being out in a shower of rain, he would pass red urine for a single micturition. Apart from this the urine was natural. The blood count was practically normal. On May 7 treatment by peptone injections was begun. The man was caused to place both his hands in ice-water for six minutes, and the urine was collected at intervals of half an hour, one, two, three, and four hours; it was still tinted with hæmoglobin after eight hours. On May 8, 10 c.c. of 5 per cent. solution of Witte's peptone was injected hypodermically. On May 9 the same cold test as before was given, but the hæmoglobinuria did not reach so severe a degree, and it lasted for only two hours.

On May 10 two further injections of 10 c.c. of the peptone solution were made, and on the 14th the cold test was negative. The urine contained no hæmoglobin, but presented a slight trace of albumin. The peptone injections were repeated upon the 15th and 18th of May, and on the 21st another negative result was obtained by the cold test. The patient then left the hospital. He returned on July 6, having had one recurrence of red urine on July 3, after a chill. On July 10 the same cold test as before—namely, immersion of the hands in ice water for six minutes—was repeated at eight o'clock, and the urine was collected at 8.30, and then at hourly intervals for twelve hours.

The axillary temperature remained normal. Hæmoglobinuria recurred and persisted for ten hours, after which it disappeared. Ten c.c. of 5 per cent. solution of Witte's peptone were injected hypodermically on July 13, 16, 19, and 22.

The cold test was repeated on the 25th at 8 o'clock in the morning; at 8.30, 15 c.c. of red urine were passed; at 9 o'clock there was still hæmoglobin present in the urine; at 10 o'clock there was a trace of albumin but no hæmoglobin; and at 11 the urine was normal.

Injections of 10 c.c. of the peptone solution were repeated on July 27 and 29, and on the 31st, after repeating the cold test, there was no hæmoglobin, though as before there was a trace of albumin.

On August 6 the cold test was repeated without any further injections of peptone having been made, and the patient stood it without any recurrence of the hæmoglobinuria; on this occasion there was not even any trace of albumin. The patient left the hospital on the sixth day, and did not return. It is very probable that he has not been cured, but there was no doubt as to the benefit he got at the time. The action of the peptone is very probably transitory, as shown by the fact that the first treatment in May, though effective at the time, was followed by a relapse in July; nevertheless the cold test was pretty severe, and had it not been for the peptone injections at the time would almost certainly have caused hæmoglobinuria upon each occasion. Even if transitory, however, the benefit of peptone treatment in this case would seem to make it worth while trying it in others of a like nature. It presents several advantages over horse serum, of which two considerable ones are the following—namely, first, that it is easier to sterilise and desterilise, if necessary; and, secondly, the injections may be repeated, as we have stated above, without any danger from anaphylaxis.

